# Predictive Value of Modified Mallampati Test and Upper Lip Bite Test Concerning Cormack and Lehane's Laryngoscopy Grading in the Anticipation of Difficult Intubation: A Cross-Sectional Study at a Tertiary Care Hospital, Bhubaneswar, India

**DOI:** 10.7759/cureus.28754

**Published:** 2022-09-03

**Authors:** Supriya Kar, Laxman K Senapati, Priyadarsini Samanta, Ganesh C Satapathy

**Affiliations:** 1 Anesthesiology, Kalinga Institute of Medical Sciences, Bhubaneswar, IND; 2 Physiology, Kalinga Institute of Medical Sciences, Bhubaneswar, IND

**Keywords:** general endotracheal anesthesia, cormack lehane classification, upper lip bite test, modified mallampati test, difficult intubation

## Abstract

Background and objective

Many tests are at hand to predict difficult intubation preoperatively to prevent morbidity and mortality of unanticipated difficult intubation. The present study was conducted to evaluate and compare the efficacy of the modified Mallampati test (MMT) and upper lip bite test (ULBT) to foresee difficult intubation.

Materials and methods

After obtaining written informed consent, this prospective comparative observational study was conducted on 225 patients scheduled for elective surgery under general endotracheal anesthesia. Preoperative MMT and ULBT were performed. MMT Grade III, IV, and ULBT Grade IV were regarded as predictors of difficult intubation. The laryngoscopic view was graded as per Cormack and Lehane's laryngoscopic grading after induction of anesthesia by an experienced anesthesiologist ignorant of preoperative airway evaluation. Patients with Cormack and Lehane Class III and IV were regarded as difficult intubation. Sensitivity, specificity, and positive and negative predictive values of MMT and ULBT were computed. Agreement between two tests with the Cormack Lehane test was determined by the Kappa coefficient.

Results

In our research, the occurrence of difficult intubation was found to be 10.2% (23 cases of difficult intubation out of 225 patients). In our analysis, we found the sensitivity (95.5% vs. 95.4%), specificity (54.8% vs 50.0%), positive predictive value (91.6% vs 93.1%), and negative predictive value (39.1% vs 39.1%) were almost comparable between modified Mallampati test and upper lip bite test. Kappa coefficient for the upper lip bite test (0.492) was slightly higher as compared to modified Mallampati scoring (0.454), but both the values are highly statistically significant (p-value <0.001).

Conclusion

Both the upper lip bite test and modified Mallampati test are comparable with each other and since the upper lip bite test is easy to perform bedside test we recommend it to be used alone or in collaboration with other tests in assessing difficult airways.

## Introduction

The basic responsibility of an anesthesiologist is the sustainment of a patent airway. Undoubtedly the leading cause of death and permanent brain damage during anesthesia is difficult or failed tracheal intubation [1]. The reported occurrence of difficult laryngoscopy and tracheal intubation ranges from 1.5% to 13% in patients undergoing general anesthesia [2]. Failure to handle difficult airways contributes to 30% to 40% of all anesthetic deaths [3]. So the need of the hour is to foresee a potentially difficult airway in the preoperative period itself to nullify such fatal events with proper prior planning.

A plethora of tests is available to predict difficult laryngoscopy and tracheal intubation such as the Mallampati test, thyromental distance, inter incisor gap, subluxation of the mandible, length of the mandibular rim, chin protrusion and atlanto-occipital extension [4]. But most of these tests are unreliable owing to their limited value in predicting difficult intubation [5-7]. In a clinical scenario test envisioning difficult intubation should possess the characteristics like simplicity, acceptability, easy performance ability along with high predictive power. The test utilized frequently is the modified Mallampatti test (MMT; modification of original Mallampatti by Samson and Young) [8-9]. The MMT has relatively high specificity but low sensitivity and a greater number of false positive results [5-6,10].

Of late, a novel technique to assess difficult intubation was proposed by Khan et al. [4], the upper lip bite test (ULBT) which was externally evaluated by Eberhart et al. [11]. They concluded that ULBT has higher specificity and predictive accuracy. The ULBT bears in mind some of the shortcomings inherent in traditional airway evaluation methods. Because of its simplicity, interobserver variability in analyzing the oropharyngeal anatomy is trivial. Although it looks promising, the amount of data available to reinforce its extensive use as the method of choice for preoperative airway assessment is meager.

Therefore, we conducted this prospective study to compare pre-operative airway evaluation methods of MMT and ULBT with Cormack-Lehane laryngoscopy grading obtained during tracheal intubation. We hypothesized that ULBT might figure out to be a good predictor of difficult laryngoscopy and intubation.

## Materials and methods

Study participants and study design

After obtaining approval from the Institutional Ethics Committee (IMS/IEC/111/2015) and receiving written informed consent, this hospital-based cross-sectional study was carried out at the Institute of Medical Sciences and SUM Hospital, a tertiary care hospital, Bhubaneswar, Odisha, India from 1st July 2015 to 31st August 2017 in patients aged 18 to 60 years with the American Society of Anesthesiologists (ASA) physical status I and II enrolled for pre-anesthetic check-ups in the anesthesiology department for surgery under general anesthesia. Patients not willing to participate in the study, those with cervical disc fracture or congenital abnormality of the neck region, those who were unable to perform the tests, those with any anatomical deformities of the neck and face, pregnant women, and those patients who cannot sit upright were excluded.

Sample size

Considering the prevalence of difficult airways to be 15% among all patients undergoing general anesthesia [12] for surgical procedures in tertiary health care centers and an allowable error of 4%, the sample size was computed using the formula, n = 4 PQ / E^2^, (Where ‘P’ is the prevalence, ‘Q’ = (100-P) & ‘E’ is the permissible error of ‘P’) [13]. So, taking ‘P’ = 15, ‘Q’ = (100-15) = 85 and ‘E’ = 5%. Therefore, n = 4 * 15 * 85 / 5^2^ = 5100 / 25 = 204, So, n=204. Adding approximately 10% of non-respondents, a total of 225 patients were analyzed for the study.

Assessment of modified Mallampati test, upper lip bite test, and Cormack and Lehane grading

Modified Mallampati grading [8] was assessed by asking the patient (in a sitting or upright position) to open his/her mouth and protrude the tongue maximally without phonation, where Class 1 = visualization of faucial pillars, soft palate, and entire uvula; Class 2 = visualization of Faucial pillars, soft palate, and uvula; Class 3 = visualization of soft palate and base of uvula; Class 4 = The soft palate is not visible, i.e. only the hard palate can be visualized at the roof of the mouth.

The upper lip bite test [4] was accomplished by asking the patient to move his/her lower jaw/teeth over the upper lip and the results were noted as follows: Class 1 = The lower incisors can bite the upper lip above the vermilion line; Class 2= The lower incisors can bite the upper lip below the vermilion line; Class 3 = The lower incisors cannot bite the upper lip.

Anesthetic technique

All patients were premedicated with injection midazolam 0.01mg/kg, injection ondansetron 0.1mg/kg and injection glycopyrrolate 0.01mg/kg, injection fentanyl 2mcg/kg. Monitors used were oxygen saturation (SpO2), ECG, non-invasive blood pressure (NIBP), end-tidal carbon dioxide (ETCO2), and additional monitors as required for each case. All the patients were induced with an injection of propofol 2mg/kg IV. If bag and mask ventilation were adequate, suxamethonium chloride 1.5mg/kg IV was given and ventilated with 100% oxygen. The head was placed in the sniffing morning air position and laryngoscopy was performed by an experienced Anesthesiologist with at least one year of experience who was unaware of the pre-operative modified Mallampati and upper lip bite classes, with a Macintosh No.3, No. 4 blade without applying external laryngeal pressure.

Cormack and Lehane Grading

The grade of glottic view according to Cormack Lehane (C-L) classification [14] (Grade I, II, III, IV) were noted where Grade I = Full view of the glottis (vocal cord visible); Grade II = Partial view of vocal cord (anterior commissure not visible); Grade III = view of the epiglottis, no vocal cord visible; Grade IV = view of the soft palate, no epiglottis visible.

Definition of outcome terms 

True positive refers to a difficult intubation that had been predicted to be difficult; false positive refers to an easy intubation that had been predicted to be difficult; true negative refers to an easy intubation that had been predicted to be easy; and false negative refers to a difficult intubation that had been predicted to be easy. Sensitivity is the percentage of correctly predicted difficult intubations as a proportion of all truly difficult intubations, i.e., true positives/(true positives + false negatives); specificity is the percentage of correctly predicted easy intubations as a proportion of all truly easy intubations, i.e., true negatives/(true negatives + false positives); positive predictive value (PPV) is the percentage of correctly predicted difficult intubations as a proportion of all predicted difficult intubations, i.e., true positives/(true positives + false positives); and negative predictive value (NPV) is the percentage of correctly predicted easy intubations as a proportion of all predicted easy intubations, i.e., true negatives/(true negatives + false negatives) [15].

Statistical analysis

Statistical Package for Social Sciences (SPSS) version 23.0 (IBM Corp., Armonk, NY, USA) was used for all analyses. The quantitative data were presented as mean±standard deviation. The unpaired sample t-test or Mann- Whitney U test was applied to compare the differences in mean values of variables between the groups wherever applicable. The qualitative data were expressed in percentages and the differences between percentages were computed using the χ2 test or Fischer exact test. The diagnostic/predictive value of tests was measured by four parameters namely sensitivity, specificity, positive predictive value, and negative predictive value. The receiver operator characteristic curve was used to evaluate the predictive efficiency of neck circumference for difficult airways. P-value <0.05 was considered statistically significant.

## Results

During the study period, we approached 239 patients undergoing pre-anesthetic check-ups for surgery under general anesthesia. Out of these patients 11 patients did not give consent or dropped out during the various procedure applied to them and five patients had incomplete data for one or more variables (dropout rate = 6.69%). Excluding these patients from the analysis, we had included a total of 225 patients in the study.

Demographic variables

The mean age of the study participants was 40.42 ± 11.88 years with the minimum age being 19 years and the maximum being 60 years. Group-wise distribution of the study participants revealed that the majority belonged to the age group between 30 to 45 years (38.2%), followed by the age group 45 years or above (36.9%) and the rest of them belonged to the age group less than 30 years (24.9%). The mean and standard deviations of height, weight, body mass index (BMI) are mentioned in Table 1.

**Table 1:** General physical examination findings of the study participants

Variables	Mean	Standard Deviation
Height (in centimeters)	157.69	6.96
Weight (in Kilograms)	59.67	12.42
Body Mass Index	30.46	1.71
Neck Circumference	23.92	4.52

Gender distribution of the study subjects showed that more than half of the participants were females (54.7%) and (45.3%) were males. The difference in body mass index between males and females was statistically significant (p-value = 0.020) as well as the neck circumference (p-value < 0.001) (Table 2).

**Table 2:** Comparison of general physical examination findings between gender groups *Independent sample t-test was used

Variable	Male (Mean ± SD)	Female (Mean ± SD)	P value*
Age (in years)	39.92 ± 12.21	40.83 ± 11.61	0.570
Body Mass Index	24.69 ± 4.02	23.28 ± 4.83	0.020
Neck Circumference (in centimeters)	31.41 ± 1.50	29.67 ± 1.46	<0.001

Clinical characteristics affecting intubation in study participants

A short muscular neck was present in 6.2% of the participants. Only 8.4% of the subjects were suffering from diabetes whereas 16.0% of the subjects were suffering from hypertension. None of the subjects were suffering from cervical spondylosis while only 4.9% of the subjects had a history of snoring.

Difficult airway according to different classification

More than half (58.7%) were classified as Grade I according to Cormack Lehane classification, followed by Grade II (31.1%), Grade III (8.4%), and Grade IV (1.8%). An almost equal percentage of patients was classified as Grade I (43.6%) and Grade II (42.7%) according to the modified Mallampati classification. Just more than one-tenth of the subjects fell under Grade III (12.0%) and the rest (only 1.8%) were classified as Grade IV according to the modified Mallampati classification. The upper lip bite test has only three tiers of classification, unlike the other two classifications. According to the upper lip bite test, 49.8% of the subject were in Grade I, followed by Grade II (37.8%), and the rest were in Grade III (12.4%) (Table 3).

**Table 3:** Grading of airway according to different classification methods

Classification system	Number	Percentage (%)
Cromack- Lahane Grading		
Grade I	132	58.7
Grade II	70	31.1
Grade III	19	8.4
Grade IV	4	1.8
Modified Mallampati scoring		
Grade I	98	43.6
Grade II	96	42.7
Grade III	27	12.0
Grade IV	4	1.8
Upper Lip Bite Test		
Grade I	112	49.8
Grade II	85	37.8
Grade III	28	12.4

Predictive value of modified Mallampati and upper lip bite test

Predictive value of a test was determined by four parameters, namely sensitivity, specificity, positive predictive value and negative predictive value. To measure predictive value, Cormack Lehane's classification was taken as the gold standard and Grade III & IV combined taken as the difficult airway whereas Grades I & II were taken as normal airways for intubation. Similarly, Grade III & IV combined were taken as the difficult airway whereas Grades I & II were taken as normal airways for intubation according to modified Mallampati scoring. With regards to the upper lip bite test, only Grade III was taken as a difficult airway whereas Grades I & II were taken as a normal airway (Table 4).

**Table 4:** Predictive value of modified Mallampati scoring and upper lip bite test with respect to Cormack Lehane classification (gold standard)

Predictive Parameters	Modified Mallampati grading	Upper lip bite test
Sensitivity	95.5 %	95.4%
Specificity	54.8 %	50.0%
Positive predictive value	91.6 %	93.1%
Negative predictive value	39.1 %	39.1%

Agreement between two tests with the Cormack Lehane test was determined by the Kappa coefficient. Kappa coefficient for the upper lip bite test (0.492) was slightly higher as compared to modified Mallampati scoring (0.454), but both the values are highly statistically significant (p-value <0.001) (Table 5).

**Table 5:** Agreement between the modified mallampati scoring and upper lip bite test with Cormack Lehane classification

	Grade III & IV		Grade I & II	Kappa coefficient	P-Value
	N (%)		N (%)		
Modified Mallampati scoring					<0.001
Grade III & IV	14 (60.9)		17 (8.4)	0.454	
Grade I & II	9 (39.1)		185 (91.6)		
Upper Lip Bite test					
Grade III	14 (60.9)		14 (50.0)	0.492	
Grade I & II	9 (39.1)		188 (93.1)		

Association of different factors with difficult airway

The mean age of a patient with difficult airways was higher as compared to a patient with normal airways which was statistically significant (p-value=0.009). Similarly, difficult airway patients had higher neck circumference as compared to normal airway patients (p-value=0.001) (Table 6).

**Table 6:** Association of the difficult airway with different parameters *Independent sample t-test was used

Variable	Normal airway (Cormack: Grade I & II) (Mean ± SD)	Difficult airway (Cormack: Grade III & IV) (Mean ± SD)	P-value*	
Age (in years)	39.72 ± 11.89	46.57 ± 10.12	0.009	
Body Mass Index	23.76 ± 4.51	25.36 ± 4.47	0.107	
Neck Circumference (in centimeters)	30.33 ± 1.59	31.56 ± 2.25	0.001	

Association of clinical findings with difficult airways

A short muscular neck was found to be significantly associated with difficult airways (p-value <0.001). Similarly, hypertension (p-value <0.001) and a history of snoring (p-value = 0.049) were statistically significantly associated with the difficult airway (Table 7).

**Table 7:** Association of clinical findings with difficult airways *Chi-squared test was used

Variable	Normal airway (Cormack: Grade I & II) N (%)	Difficult airway (Cormack: Grade III & IV) N (%)		P-value*
Short muscular neck			<0.001	
Present	7 (3.5)	7 (30.4)		
Absent	195 (96.5)	16 (69.6)		
Diabetes			0.422	
Present	16 (7.9)	3 (13.0)		
Absent	186 (92.1)	20 (87.0)		
Hypertension			< 0.001	
Present	26 (12.9)	10 (43.5)		
Absent	176 (87.1)	13 (56.5)		
History of Snoring			0.049	
Present	8 (4.0)	3 (13.0)		
Absent	194 (96.0)	20 (87.0)		

Association of the number of attempts for intubation with the type of airway

More than one attempt for intubation was higher (52.2 %) in patients who are classified as having a difficult airway by Cormack and Lehane classification as compared to only 0.5% in normal airway patients. This difference was statistically significant (p-value <0.001).

Association of neck circumference with difficult airway

**Figure 1:**
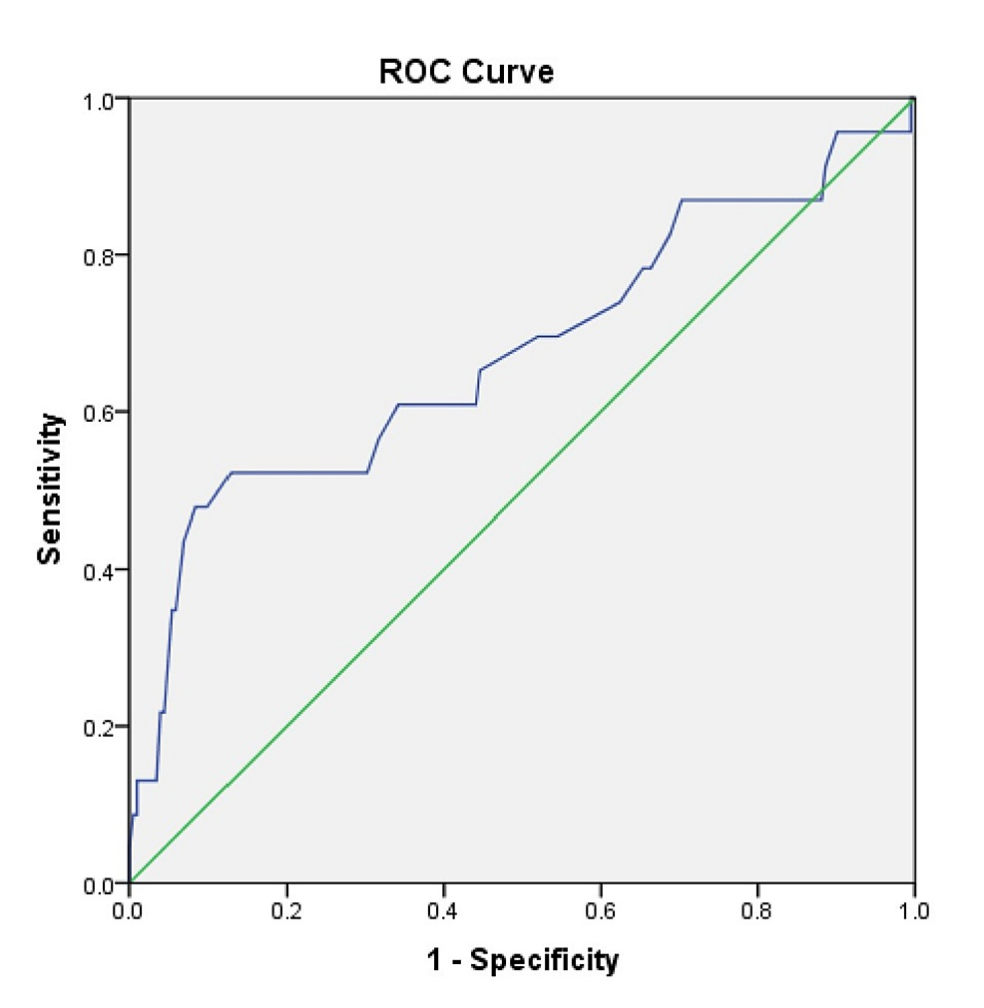
Receiver operative characteristic (ROC) curve for the neck circumference in predicting difficult airway

Finally, as a secondary objective of the study, we also tried to find out the predictability of neck circumference for the determination of difficult airways. Figure 1 shows the receiver operative characteristic (ROC) curve for the same and the area under the curve i.e. 0.675 which shows that neck circumference can be a predictor of the difficult airway (p-value = 0.006) (Figure 1).

## Discussion

In the contemporary practice of anesthesia, unanticipated difficult endotracheal intubations are the leading causes of anesthesia-related morbidity and mortality [16-17]. Therefore, it is key to foresee difficulty in laryngoscopy and intubation to get prepared with all state-of-the-art gadgets or new substitute techniques for securing the airway. Difficult intubation depends on a variety of factors and hence a plethora of predictive tests are at hand either alone or in combination [16]. Correspondingly, it is crucial to recognize a clinical test that is brisk and easily accomplished during the time of preoperative evaluation for the sake of predicting potentially difficult endotracheal intubations with high sensitivity and specificity and high PPV with few negative predictions. Accordingly difficult cases are not overlooked and detrimental life-threatening episodes are evaded.

The ULBT by Khan and his colleagues [4] was an attempt as an alternative to the most widely used modified Mallampati test. ULBT evaluates jaw movement, presence or absence of bucked teeth, and ability to protrude lower jaw. They ascertained that ULBT was easy to execute within seconds of demonstrating it to the patients and very suitable to perform as a bedside test.

In our research, the occurrence of difficult intubation was found to be 10.2% (23 cases of difficult intubation out of 225 patients) which is comparable to the results obtained by Cook et al. [18] and Savva et al. [19]. Nevertheless, the documented incidence of difficult laryngoscopy or intubation is 1.5% to 8% [20]. The contrasting anthropometric features among populations and the criteria that are used to delineate difficult intubation leads to such a wide variation in the incidence. We successfully intubated all the patients in our study. In our analysis, we found the sensitivity (95.5% vs. 95.4%), specificity (54.8% vs 50.0%), PPV (91.6% vs 93.1%), and NPV (39.1% vs 39.1%) were almost comparable between MMT and ULBT.

Khan et al. compared the ULBT and MMT and showed that the former was more accurate (88.7% versus 66.8%), while sensitivity, NPV, and PPV were similar in both tests [4]. A study conducted by Sinharay et al. [21] observed that sensitivity (88.46%), PPV (71.87%), and NPV (97.45%) of ULBT were higher than MMT with similar specificity of both tests. Likewise, a study conducted by Ali et al. [22] revealed that the sensitivity (87.5%), PPV (71.6%), and NPV (97.3%) of ULBT were higher than MMT with comparable sensitivity.

In contrast, a study carried out by Hester et al. [15] ascertained that ULBT was superior to MMT in all aspects like sensitivity, specificity, PPV, and NPV. Koirala et al. figured out that the sensitivity, specificity, PPV, and NPV of ULBT were 50%, 100%, 100%, and 91% respectively which were significantly higher compared to MMT or thyromental distance (TMD) or MMT and TMD combined [23].

The sensitivity of ULBT in our study is higher than in many previous studies [11,24-26]. The potential explanations are a paucity of inter-observer variation in our study in addition to ethnic differences. The anthropological literature well substantiates human ethnic craniofacial variation, and the dental literature validates significant racial variation in mandibular and maxillary morphology and morphometry [27-29].

The specificity of MMT in our research is around 50% which is in accordance with the finding of Khan et al. (66.8%) [4] and Eberhart et al. (61%) [11]. A handful of studies also revealed a higher specificity compared to our study like Cattano et al. [30]. We have found the sensitivity of MMT was 95.5% which was more than that of Erzi et al. [31] (76%), Schmitt et al. [32] (76%), and Eberhart et al. [11]. The sensitivity of MMT varies from 34% to 66% according to Lee et al. [10] and 0% to 100% as stated by Lundstrom et al. [33].

To ascertain the appropriate level of classification as per MMT, one needs to be cognizant of the complex oropharyngeal anatomy. The improper assessment of the test, the intricacy involved in the demonstration, and inter-observer flexibility seen in MMT may be the reason behind the wide variations documented in specificity and sensitivity in various pieces of research as was also found by Eberhart et al. [11]. Oates et al. [34] discovered that maximal extrusion of the tongue and mouth opening is the key factor in attaining a reliable MMT score. Involuntary phonation amid the test thereby altering MMT classification eventually leads to a low predictive value of MMT as discerned by Bilgin et al. [35].

The positive predictive value of MMT in our analysis was 91.6% which is sky-high in contrast with many other studies. One possible explanation for this might be the aspect that MTT was conducted by the primary investigator, thereby limiting the risks of interobserver discrepancy.

Limitations

The drawback of our study is that occasionally patients fail to understand completely and stick to the instructions as is true for MMT which can be lessened by proper demonstration of the test before the patient by the anesthesiologist. To carry out ULBT patients need a persistent illustration and four patients misapprehend despite continuous demonstration and they were excluded from our study. The other limitation of ULBT is that it can't be executed in edentulous patients, patients with limited mouth opening along with non-cooperative patients. Another shortcoming includes the fact that it does not assess neck mobility which is one more principal factor in predicting difficult intubation.

## Conclusions

Sensitivity, specificity, positive predictive value, and negative predictive value were comparable between the UBLT and the MMT. Agreement between the two tests with Cormack Lehane's test was determined by Kappa coefficient which was slightly higher for the UBLT (0.492) as compared to modified Mallampati scoring (0.454), but both the values are highly statistically significant. To conclude, the UBLT and the MMT are comparable and since the UBLT is an easy-to-perform bedside test we recommend it to be used alone or in collaboration with other tests in assessing difficult airways. Additional research is desired on ULBT with the inclusion of patients with higher ASA grades belonging to divergent ethnicities, emergency cases, obese patients with BMI≥ 35kg/m², pregnant patients, and edentulous patients.

## Appendices

Author Contribution: Study Design: Supriya Kar, Laxman K. Senapati. Study Registration: Supriya Kar. Patient Recruitment and Data Collection: Supriya Kar, Laxman K. Senapati. Clinical Operation: Supriya Kar, Laxman K. Senapati, Ganesh C. Satapathy. Manuscript Writing and Statistical Analysis: Laxman K. Senapati, Priyadarsini Samanta.
